# Acephalgic retinal migraine: A 6-year follow-up case report and literature review

**DOI:** 10.1097/MD.0000000000047894

**Published:** 2026-03-06

**Authors:** Abdullah Alhewiti

**Affiliations:** a Department of Family and Community Medicine, Faculty of Medicine, University of Tabuk, Tabuk, Saudi Arabia.

**Keywords:** headache, ophthalmic migraine, retinal migraine, retinal vasospasm, transient monocular visual loss

## Abstract

**Rationale:**

Retinal migraine is a rare cause of transient monocular visual loss that presents a significant diagnostic challenge and necessitates the careful exclusion of alternative etiologies. This case report describes the 6-year clinical course of a rare, headache-free variant of retinal migraine.

**Patient concerns:**

A 42-year-old male experienced recurrent transient monocular visual loss episodes 2 to 3 times per year, affecting either eye, without any associated headache. Each episode involved stereotypical visual symptoms starting with photopsia, followed by an expanding altitudinal visual field defect.

**Diagnoses:**

Extensive investigations, including neuroimaging, vascular studies, blood work, and ophthalmological examinations, returned normal results. After years of investigations and excluding other etiologies, a diagnosis of retinal migraine was established based on the International Classification of Headache Disorders 3rd edition criteria.

**Interventions:**

The patient opted for low-dose aspirin to decrease the risk of permanent visual loss.

**Outcomes:**

The patient continued to experience similar visual episodes without any change in frequency, severity, or characteristics.

**Lessons:**

Retinal migraine may occur without headache, consistent with current International Classification of Headache Disorders 3rd edition criteria. Careful history-taking remains a cornerstone of accurate diagnosis, and time can serve as an invaluable diagnostic tool. Finally, a patient-centered approach is essential, particularly when evidence-based management options are limited.

## 1. Introduction

Retinal migraine is a rare entity that can cause transient monocular visual loss (TMVL). It is characterized by repeated transient attacks of monocular positive and/or negative visual phenomena that may be followed or accompanied by headache.^[1]^ In contrast, the visual aura of a typical migraine is bilateral and originates from the occipital cortex.

Recent developments in diagnostic criteria have significantly impacted the recognition of retinal migraine. The prior criteria of retinal migraine in the International Classification of Headache Disorders 2nd edition, required a migraine headache to occur within 60 minutes of the visual symptoms. However, the current definition in the International Classification of Headache Disorders 3rd edition (ICHD-3) adds more detail to the symptoms and does not mandate headache accompaniment. The attacks must fulfill the criteria of a migraine with aura. In addition, they must have at least 2 of the following features: an evolving pattern of visual symptoms over ≥5 minutes, symptoms lasting 5 to 60 minutes, or accompanied or followed by headache within 60 minutes of the symptoms’ onset.^[2]^ Current understanding regards retinal migraine as a diagnosis of exclusion, necessitating the careful elimination of the more common alternative etiologies of TMVL.^[3]^ Contemporary management approaches emphasize individualized treatment, given the limited evidence for specific interventions.^[4]^

Retinal migraine remains poorly characterized in its acephalgic (headache-free) variant. This report details a 6-year clinical course of recurrent TMVL, illustrating the diagnostic journey of acephalgic retinal migraine and therapeutic considerations.

## 2. Case presentation

A 42-year-old Middle Eastern male, working as a computer programmer, presented to the family medicine walk-in clinic in 2019 with a history of sudden visual loss in his left eye, which lasted for about 10 minutes on the morning of the clinic visit. He had experienced 2 previous attacks, which were initially attributed to stress or visual strain. When he noticed that these attacks tended to recur in a similar pattern in both eyes, he became worried and visited our clinic. Upon taking further medical history, he did not report any chronic medical illnesses and was not on medication. He had no numbness or weakness, and there was no history of headache, eye pain, convulsion, confusion, or trauma. On physical examination, there were no signs of lateralization or carotid bruit. The eye exam was unremarkable, with 20/20 visual acuity, normal movement, and no visual field or relative afferent pupillary defects. He was suspected of having a transient ischemic attack (TIA) and referred to the emergency room (ER) for urgent investigation. In the ER, he had a normal electrocardiogram and computed tomography scan, and he was referred to the ophthalmology clinic for follow-up. In the ophthalmology clinic, his retinal examination and fluorescein angiogram were normal. Complete blood count, erythrocyte sedimentation rate, and C-reactive protein tests were also normal. Later, he was seen by a neurologist, and both an electroencephalogram and a visual evoked potential test were normal. Further tests for antinuclear antibodies, antiphospholipid antibodies, and other hypercoagulable workups were negative. Magnetic resonance imaging, magnetic resonance angiography, and echocardiography were normal. Since the initial visit, he underwent multiple repeated investigations, all of which were normal. This led the neurologist, 2 years later, to diagnose him as having a typical migraine aura without headache and advised him to continue to follow up with family medicine clinics without the need for medications.

**Figure 1.**
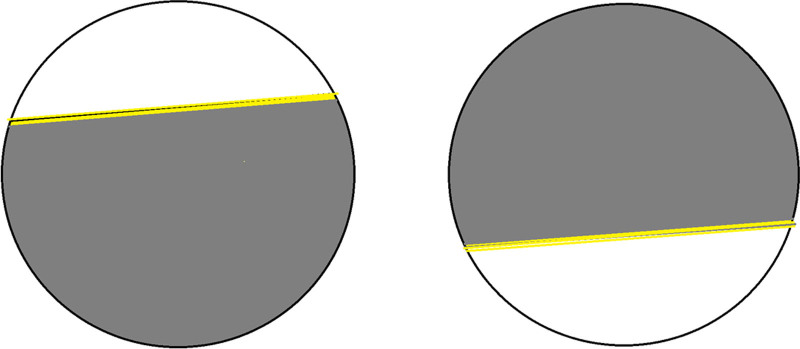
Patient’s illustration of his monocular visual phenomena as an ascending or descending gray visual field defect with a scintillating advancing edge.

Subsequently, the patient continued to visit our clinics in-person twice a year. After 2 years under our care, the patient’s ongoing concern about his symptoms prompted a more thorough reevaluation of his entire medical history. He reported persistent episodes occurring 2 to 3 times per year and emphasized that they were strictly monocular, affecting either the right or the left eye. These episodes resolved spontaneously and never lasted more than 10 minutes. He believed that stress precipitated these events, although he never had a headache during these episodes. The patient described these attacks as beginning abruptly with a “large, bright light area in his visual field” (photopsia) that gradually resolved into “a gray-shaded zone” (scotoma) with “a yellow-orange colorful edge” (scintillation). This scotoma progressively expanded until it covered most of his vision, spreading in an altitudinal pattern – sometimes ascending from the lower visual field, and other times descending from the upper visual field. He also described a separate phenomenon of seeing colorful, wavy, curved lines (fortification spectra) in either eye, which disappeared gradually without evolving into visual loss. He reported that these episodes seemed distinct and unrelated to his typical monocular attacks. He also had a positive family history of severe migraine; however, he did not consider himself a migraineur. The patient was asked to illustrate his visual phenomena using a colored figure, which he provided as in Figure 1.

After a thorough reevaluation of the patient’s history, it was clear that the patient did not exhibit a cortical pattern of visual phenomena. Given the recurrent TMVL episodes over a prolonged period, and after the exclusion of alternative etiologies, the diagnosis of retinal migraine was established in accordance with the ICHD-3 criteria (Table 1). The diagnosis was challenging because acephalgic retinal migraine is extremely rare, lacks a headache, and mimics more common, serious causes of TMVL like thromboembolic disease (amaurosis fugax/TIA).

**Table 1:** Patient presentation mapped with International Classification of Headache Disorders 3rd edition (ICHD-3) criteria.

ICHD-3 criterion	Patient’s presentation	Supporting evidence
Attacks fulfilling criteria for migraine with aura	Multiple attacks of reversible aura (retinal), which have the following characteristics (gradual ≥ 5 min, lasting > 5 min, unilateral and positive)	Patient illustration and detailed description
Fully reversible, monocular, positive and/or negative visual phenomena confirmed by either/both:	Multiple episodes over 6 yr (2 and 3/yr)	Patient’s drawing
Visual field examination during the attack		
Patient’s drawing		
At least 2 of the following 3:	Fulfilled	2 out of 3
Evolving pattern over ≥5 min	Photopsia followed by a progressive visual field defect. Gradual progression ≥5 min	Detailed patient characterization
Duration 5–60 min	Episodes consistently last >5 min (8–10 min)	Consistent patient reporting
Accompanied/followed by headache within 60 min	Headache absent	ICHD-3 allows headache-free presentation
Not better accounted for by another disorder	Extensive workup excluding alternatives	Waited for 4 yr before labeling as retinal migraine following repeated stereotypical visual phenomena and negative workup findings

The patient was informed about the possibility of having undetected vascular pathology and counseled on the potential risk of permanent visual loss (PVL) associated with retinal migraine according to the best available evidence. After weighing the benefits and risks of different interventions, the patient chose to start taking low-dose aspirin (acetylsalicylic acid 81 mg/day). The patient did not prefer to start verapamil or other calcium channel blockers (CCBs) as preventive therapy for the infrequent, short-lived episodes of retinal migraine. He also expressed concerns about the difficulty with adherence and the side effect profile of CCBs because of his family history with migraine prophylaxis. In addition, he declined the use of antiepileptics and tricyclic antidepressants for the same reasons. He was advised to visit the ER if an unusually prolonged episode developed. He was also informed about the need to revisit his management plan in case the severity of his symptoms changes.

For the last 2 years following initiation of medication, the patient attended regular follow-up visits to reevaluate the benefits and risks of aspirin therapy, and to monitor adherence and progression. The adherence and potential progression were assessed by patient self-report and in-office visual acuity screening using a Snellen chart. Aspirin tolerance was evaluated by a history of gastrointestinal upset at every visit and by annual follow-up of hemoglobin levels. In March 2025, the patient reported that he remained adherent to the daily aspirin, with no change in his visual symptoms frequency or severity. Table 2 summarizes the course of the case over the years.

**Table 2:** Diagnostic journey, management, and follow-up for a case of retinal migraine.

Year	Event	Investigation/intervention	Result/outcome
2019 (initial visit)	Left eye TMVL, <10 min. History of 2 previous attacks	Emergency evaluation: ECG, CT head	Normal results, suspected TIA
2019	Ophthalmology referral	Retinal exam, fluorescein angiogram, CBC, ESR, CRP	All normal
2019	Neurology consultation	EEG, VEP testing	Normal results
2019–2021	Extended workup	MRI, MRA, echocardiography, hypercoagulable studies, ANA, ANCA, RF, anti-CCP, HIV, hepatitis B and C serology, syphilis serology, QuantiFERON-TB Gold, TB screening, OCT, repeated CBC, ESR, CRP, and metabolic panels	All normal
2021	Initial diagnosis	Clinical assessment	Diagnosed as a typical aura without headache
2021–2023	Regular follow-up	Twice-yearly in-person family medicine visits, with symptom tracking and routine health maintenance	Continued episodes 2 and 3/yr, no progression
2023	Detailed history revisit	Comprehensive symptom characterization with patient illustration, repeated blood work. ICHD-3 criteria application	Retinal migraine diagnosed
2023	Preventive measure of PVL	Low-dose aspirin 81 mg daily	Patient choice after comprehensive counseling
2023–2025	Treatment follow-up	Biannual in-person monitoring visits	Stable course, visual acuity at 20/20 OU (2020–2025); tolerant to aspirin with no change in the visual symptoms

ANA = antinuclear antibodies, ANCA = antineutrophil cytoplasmic antibodies, including p-ANCA (perinuclear ANCA) and c-ANCA (cytoplasmic ANCA), anti-CCP = anti-cyclic citrullinated peptide, CBC = complete blood count, CRP = C-reactive protein, CT = computed tomography, ECG = electrocardiogram, EEG = electroencephalogram, ESR = erythrocyte sedimentation rate, HIV = human immunodeficiency virus, ICHD-3 = International Classification of Headache Disorders 3rd edition, MRA = magnetic resonance angiography, MRI = magnetic resonance imaging, OCT = optical coherence tomography, OU = oculus uterque, PVL = permanent visual loss, QuantiFERON-TB Gold = quantitative interferon gamma release assay-TB, RF = rheumatoid factor, TIA = transient ischemic attack, TMVL = transient monocular visual loss, VEP = visual evoked potential.

## 3. Discussion

This is the first case report of acephalgic retinal migraine with a prolonged, 6-year regular follow-up, enriched by the patient’s own illustrated description of his visual phenomena. This case is also unique in demonstrating how time itself can serve as a diagnostic tool after extensive negative investigations. The extended observation shows that retinal migraine can present consistently without headache, per ICHD-3 criteria, and maintain a stable clinical course. Unlike previous reports focusing on acute presentations, this case provides valuable long-term prognostic data and highlights the diagnostic challenges of the headache-free variant of retinal migraine.

TMVL is a complaint that is relatively uncommon in family medicine clinics. These patients need urgent evaluation for possible serious etiologies and to manage them accordingly to prevent severe complications.^[5]^ Causes of TMVL include, but are not limited to, TIA/amaurosis fugax, giant cell arteritis/vasculitides, optic neuritis, multiple sclerosis, retinal detachment, and intermittent angle-closure glaucoma.^[5]^ The most common cause of TMVL is amaurosis fugax resulting from an embolic event due to carotid or cardiac pathology.^[6]^ A careful history, physical examination, and comprehensive workup are essential for accurate diagnosis. The workup should include erythrocyte sedimentation rate, C-reactive protein, electrocardiogram, electroencephalogram, cardiac echocardiography, neuroimaging, and vascular studies. All cases of TMVL should have a detailed fundoscopic examination by an ophthalmologist.^[7]^ A consultation with a neurologist may be necessary, depending on the findings, to rule out other possible neurological etiologies. A secondary cause is unlikely to be found if the TMVL recurs in a similar pattern for a long period of time.^[8]^ Table 3 lists important differential diagnoses and reasons for exclusion in the current case.

**Table 3:** Differential diagnoses of transient monocular visual loss and reasons for exclusion in the current case.

Differential diagnosis	Reasons for exclusion in this case
Amaurosis fugax/TIA	Normal MRA, ECG, echocardiogram, and hypercoagulability workup. Episodes were stereotyped and lasted for years without any permanent deficit, which is atypical for embolic events.
Giant cell arteritis	Age, no headache, no systemic symptoms. ESR, CRP, and MRA were normal.
Other vasculitides	Comprehensive serological testing was negative (ANA, ANCA, APL, RF, anti-CCP, viral). No systemic manifestation.
Retinal detachment	Ophthalmological exams and OCT were normal.
Intermittent angle-closure glaucoma	No pain, halos, or redness. Normal ophthalmological exams.
Multiple sclerosis/chronic optic neuropathy (Uhthoff phenomenon)	No other neurological signs or symptoms. No optic neuritis. Episodes were not triggered by heat or exertion. Normal VEP and MRI make demyelination highly unlikely.
Optic neuritis	Ophthalmological exams, VEP, and OCT were normal. No pain with eye movement.
Optic disc drusen	Ophthalmological exams and OCT were normal. The course of illness makes it unlikely.
Papilledema	Ophthalmological exams and OCT were normal. No high ICP or headache. Symptoms lasted 10 min, not seconds.
Orbital mass	MRI and CT were normal. Symptoms were not gaze-evoked and affected either eye.
Sickle cell anemia	Normal CBC. No childhood history.
Coagulopathy/hypercoagulable states	Negative hypercoagulable workup. No personal or family history of thrombosis.
Migraine aura (typical/cortical)	Indeed, the patient reported that he was initially confused in the first few attacks whether they were monocular or binocular. However, with a meticulous review of the patient’s history, we confirmed that the patient’s symptoms were strictly monocular.
Drug-induced vasospasm/stimulant abuse	The patient was not on medications and had no history of illicit drug use (can mimic retinal migraine).
Conversion disorder	No history of psychological stressors or mental disorders. The presentation and pattern of TMVL are unlikely due to conversion disorder.
Other rare retinal or optic nerve disorders (e.g., leber hereditary optic neuropathy, anterior ischemic optic neuropathy, subtle autoimmune retinopathy)	Normal ophthalmological examination. Also, a recurrent stereotypical pattern and stability for years make it extremely unlikely in these cases.

ANA = antinuclear antibodies, ANCA = antineutrophil cytoplasmic antibodies, anti-CCP = anti-cyclic citrullinated peptide, APL = antiphospholipid antibodies, CBC = complete blood count, CRP = C-reactive protein, CT = computed tomography, ECG = electrocardiogram, ESR = erythrocyte sedimentation rate, GCA = giant cell arteritis, ICP = intracranial pressure, MRA = magnetic resonance angiography, MRI = magnetic resonance imaging, OCT = optical coherence tomography, RF = rheumatoid factor, TIA = transient ischemic attack, TMVL = transient monocular visual loss, VEP = visual evoked potential.

The pathophysiology of retinal migraine likely involves retinal vasospasm. This has been demonstrated by direct photographic evidence in a report by Doyle et al^[9]^ and angiographically by Kosmorsky and others.^[10,11]^ The current case’s response to stress triggers, family history of migraine, and characteristic visual phenomena support a migrainous mechanism. The absence of headache in this case aligns with the ICHD-3 acknowledgment that such presentations occur, though the classification notes that migraine etiology cannot be definitively established in these instances. However, the combination of prolonged stereotypical symptoms, family migraine history, stress triggers, and exclusion of alternative etiologies provides compelling evidence for retinal migraine as the underlying diagnosis. It is important to note that 1 condition that may mimic retinal migraine is retinal vasospasm due to stimulant abuse.^[12]^

Compared to previously published retinal migraine case reports over the past 25 years (Table 4), the current case exhibits several distinctive features that highlight its unique contributions.^[3,9–11,13–21]^ In terms of headache association, this case joins only 4 other reports (Doyle et al,^[9]^ Kosmorsky,^[10]^ Ashfaq,^[19]^ and Chhabra et al^[20]^) in documenting acephalgic presentations. These cases represent approximately 20% of published cases in the last 25 years and support the validity of headache-free retinal migraine as recognized by ICHD-3 criteria. Regarding the follow-up duration, the current case provides the longest documented regular follow-up period of acephalgic migraine. In Kosmorsky’s case, the patient was known to have menstrual migraine and developed an attack of retinal migraine in 1988. There was no clear documentation of follow-up or specification of treatment duration. The patient was contacted in 2011 and reported no further episodes since the documented attack.^[10]^ The follow-up period of other cases of retinal migraine ranges from 1 month to 10 years. Treatment approaches varied considerably across reports. While Grosberg et al^[15]^ employed various prophylactic agents, Gan et al^[13]^ and Chhabra et al^[20]^ used aspirin in headache-associated cases. Most critically, regarding PVL outcomes, the current case demonstrates a remarkably favorable prognosis with no progression to PVL over 6 years. This contrasts with the concerning 16.7% in Grosberg et al^[15]^ cohort and the devastating permanent losses documented by Gutteridge et al^[16]^ and Chhabra et al^[20]^ (accounting for 20% of included cases in Table 4). This suggests that either acephalgic variants may have a more benign course, or early aspirin intervention may be protective.

**Table 4:** Summary of case reports and series on retinal migraine published since 2000.*

Study/author	Year	N	Age (yr)	Gender	Associated headache	Clinical features	Follow-up duration	Treatment	Permanent visual loss	Key findings
Doyle et al^[9]^ (case report)	2004	1	22	M	No	Recurrent left eye TMVL (~10 min). During episode: fundoscopy showed arterial/venous constriction, disc pallor, cherry red spot, NLP.	NR	None	No	Photographic evidence of reversible retinal vasoconstriction. Workup negative, supporting primary vasospasm.
Gan et al^[13]^ (case report)	2005	1	40	M	Yes	Recurrent TMVL since adolescence (~5–10 min), gray spots, monocular. Migraine headache followed visual symptoms.	NR	Aspirin daily	No	Treatment with aspirin and verapamil reduced episode frequency from daily to weekly.
								Verapamil daily		
Grosberg and Solomon^[14]^ (case report)	2006	2	Case 1: 30	F	Case 1: associated with most episodes. One major episode was without a headache.	Both with a history of migraine with typical aura who experienced recurrent, prolonged (hours to weeks), but fully reversible, monocular scotoma or blindness.	Case 1: 7 yr	Multiple prophylactics tried (CCBs, β-blockers, antidepressants, AEDs, etc) with inconsistent benefit. Acute attacks treated with opioids (case 1), triptans/ergots (initially, case 2).	No	Demonstrates that monocular visual auras can be prolonged (hours to weeks) yet still fully reversible. Challenges the ICHD-2 duration limit and suggests “monocular migraine” is a more accurate term than “retinal migraine.”
			Case 2: 41		Case 2: yes		Case 2: 10 yr			
Grosberg et al^[15]^ (case series/review)	2006	Six new cases	Mean ~35.3 (onset range: 7–43)	5F, 1M	Yes (100%)	Recurrent, fully reversible TMVL (5 min–3 d) ipsilateral to migraine headache (4 MA, 2 MO). One case had associated seizures.	6–10 mo	Prophylaxis: topiramate, nortriptyline, verapamil, Mg/riboflavin, aspirin.	1/6	Modern prophylaxis was highly effective in preventing TMVL and headache. One case of PVL was largely reversed with treatment.
Gutteridge et al^[16]^ (case report)	2007	1	60	M	Yes	A history of MA presented with a persistent inferior arcuate scotoma in his right eye. The event began with a shimmering, gray scotoma lasting 30–40 min, followed by a headache. Fundoscopy revealed nerve fiber layer opacification consistent with a BRAO.	18 mo	Aspirin	Yes	Presents a rare case of retinal migraine complicated by a permanent BRAO attributed to protracted vasospasm associated with a migraine attack. Suggests that in acute presentations, calcium channel blockers and aspirin may be of therapeutic value.
Kosmorsky^[10]^ (case report)	2013	1	30	F	No headache during visual episodes	Eight to ten episodes of right eye TMVL over 3 d (flashing lights, gray-outs, NLP). Associated with anorexia/abdominal tenderness. History of menstrual migraine without aura.	Called 2011 after the 1988 episode. No clear regular follow-up	None reported for episodes; prior migraines were untreated.	No	Angiographically documented central retinal artery vasospasm during an attack with complete resolution. Twenty-three years later, reported no other episode.
Ota et al.^[17]^ (case report)	2013	1	29	F	Yes	Ten-year history of MA recurrent TMVL “whiteout” in the right eye, lasting 2–5 min.	NR	Propranolol hydrochloride	No	First fundus video documentation of dynamic retinal vasospasm during an attack. Attacks ceased with prophylaxis.
El Youssef et al^[18]^ (case report)	2018	1	59	M	Yes	Five episodes of left eye TMVL over 2 d, each lasting ~5 min, described as a “curtain moving” nasally to temporally.	NR	Aspirin and valproate	No	Fundus photography showed multifocal CRA/CRV vasoconstriction with complete resolution 10 min later.
Ashfaq^[19]^ (case report)	2020	1	26	F	No	Seven and eight episodes of right eye blindness over 30 h, each lasting 5–7 min. Described as a “white curtain” moving from the inner to the outer aspect. History of migraine.	1 mo	Reassurance and counseling only. No medication.	No	Presents a headache-free variant of retinal migraine. The progression of vision loss from the inner (nasal) aspect is noted as unusual.
Maher et al^[3]^ (case report/review)	2021	One new case	41	F	Yes	History of migraine. Onset of the right. Gradual blurry vision progressing to complete “blackout” lasting 3–7 min. Associated nausea.	Not explicitly stated (referred to clinic “several years after onset”)	Preventive: Mg citrate, riboflavin daily, melatonin	No	A regimen of Mg, riboflavin, melatonin, and rizatriptan was successful in reducing the frequency and severity of both retinal migraines and typical migraine headaches.
								Acute: rizatriptan		
Chhabra et al^[20]^ (case report)	2021	2	Case 1: 57	M	Case 1: no	Case 1: history of retinal migraine. Presented with persistent monocular vision loss that progressed over 4 d from blurry vision to finger-counting. Diagnosed with PAMM progressing to CRAO.	Case 1: 12 mo	Case 1: acute sublingual nitroglycerin, lasmiditan, naproxen. Preventive: aspirin, atorvastatin, nortriptyline, Mg, verapamil.	Yes	Retinal migraine can be complicated by PVL due to ischemic infarction, CRAO, ION. This suggests a spectrum of “migrainous infarction” affecting the retina or optic nerve. Contraindicates the use of triptans. Suggests acute use of vasodilators (nitroglycerin) and anti-inflammatories/antiplatelets (NSAIDs, aspirin). Proposes that ICHD-3 criteria could be expanded to include these irreversible ocular complications.
			Case 2: 27		Case 2: yes	Case 2: history of 1 prior retinal migraine. Presented with a persistent right eye superotemporal scotoma following a scintillating scotoma and headache. Diagnosed with posterior ION.	Case 2: 1 mo	Case 2: acute: naproxen and aspirin at aura onset. Preventive: Mg citrate, melatonin.		
González-Martín-Moro et al^[11]^ (case report)	2023	1	27	M	Yes	Patient had a history of migraine with aura, presented with a left-sided headache and a new, persistent “bluish stain” in the center of his right eye.	1 yr	Naproxen (at home), intravenous metamizol.	No	First OCT-angiography documentation of retinal hypoperfusion. Demonstrates that these vascular changes are fully reversible and do not lead to permanent structural damage.
Pereira et al^[21]^ (case report)	2024	1	41	M	Yes	Twenty-four-hour persistent L eye scotoma following a typical aura/headache.	1	Naproxen	No	Demonstrates that a migraine attack can cause retinal capillary ischemia, evidenced by SD-OCT. Vision returns to normal after 1 mo with no permanent damage. Emphasizes the need for multimodal retinal imaging.

AEDs = antiepileptic drugs, BRAO = branch retinal artery occlusion, CCBs = calcium channel blockers, CRA = central retinal artery, CRAO = central retinal artery occlusion, CRV = central retinal vein, ICHD = International Classification of Headache Disorders, ION = ischemic optic neuropathy, MA = migraine with aura, Mg = magnesium, MO = migraine without aura, NLP = no light perception, NR = not reported, NSAIDs = nonsteroidal anti-inflammatory drugs, OCT = optical coherence tomography, PAMM = paracentral acute middle maculopathy, PVL = permanent visual loss, SD-OCT = spectral domain optical coherence tomography, TMVL = transient monocular visual loss.

* One case report was not included in the table because of a full-text access issue.

In comparison with previous reviews, Grosberg et al^[15]^ conducted the most comprehensive analysis to date, examining 46 cases (40 from the literature review plus the 6 new patients) in 2006. Disturbingly, they reported that 21 patients (46%) developed PVL. These findings challenged the notion that retinal migraine represents a benign condition.^[15]^ In 2007, Hill and colleagues^[22]^ conducted another review and applied more stringent International Classification of Headache Disorders 2nd edition criteria. They included only 16 patients and argued that Grosberg et al overdiagnosed retinal migraine and that most of the cases may represent a vascular etiology.^[22]^ Nevertheless, subsequent case reports have continued to document PVL, including 2 recent cases in 2021.^[20]^ This accumulating evidence suggests that, irrespective of the specific diagnostic criteria used, retinal migraine is not an entirely benign entity and is associated with a measurable risk of PVL.^[8]^

There is no definitive evidence-based treatment for retinal migraine. For patients with recurrent troublesome symptoms, preventive therapy with tricyclic antidepressants, antiepileptics, or CCBs may be beneficial.^[8]^ For instance, CCBs like verapamil or nifedipine are often considered a first-line preventive option due to their vasodilatory effects, which may counteract the retinal vasospasm.^[23]^ On the other hand, the use of tricyclic antidepressants and antiepileptics is extrapolated from their efficacy in other types of migraine, and they can be considered as alternatives for patients with troublesome symptoms (e.g., headache and frequent attacks). However, the evidence for antiepileptics and tricyclic antidepressants is weaker, especially for the acephalgic variant, and they have no well-established direct effect on retinal vasospasm. Medications that may worsen vasoconstriction, such as triptans, ergot alkaloids, and beta-blockers, should be avoided, as should medications that promote hypercoagulability, such as oral contraceptive pills.^[8,24,25]^ For an acute prolonged episode, emergency management with oxygen and initiation of a vasodilating agent such as verapamil or nifedipine is a reasonable approach.^[23,26]^

The main challenge in managing retinal migraine is the prevention of PVL. Although strong evidence is lacking, primarily driven by previous case reports and some recommendations, low-dose aspirin can be used to decrease the risk of PVL due to an occlusive event.^[8,13]^ The theoretical rationale for antiplatelet therapy in preventing retinal vascular occlusion is to reduce the risk of microthrombi formation at the site of vasospasm. This management option differs from that of other types of migraine, where aspirin is not usually used as a primary prevention for ischemic events. This difference is based on the much higher risk of PVL associated with retinal migraine when compared to the risk of migrainous stroke linked to other types of migraines.^[8]^

This single case report, while providing valuable long-term data, has inherent limitations in terms of generalizability. Treatment approaches should be individualized in each case, and patients should be informed about the available treatment options and the alternatives. In this case, the patient’s choice of aspirin was guided by its favorable safety profile and the theoretical benefit, while CCBs were declined due to concerns about adherence, side effects, and the infrequency of his episodes. Furthermore, we cannot completely rule out the possibility of a subtle vascular or autoimmune pathology. This remains a remote possibility, even though it is extremely unlikely given the prolonged period without any other manifestations or progression.

## 4. Conclusion

This rare case provides the longest documented regular follow-up of acephalgic retinal migraine, validating the current ICHD-3 criteria. The 6-year course without PVL challenges existing prognostic assumptions and suggests that acephalgic variants may have more benign outcomes than previously recognized. While advanced diagnostic technologies are fundamental to modern medicine, this case underscores the critical role of meticulous history-taking and effective communication skills in achieving an accurate diagnosis. It also emphasizes the importance of dealing with uncertainty in clinical practice, utilizing time as a valuable diagnostic tool, and adopting a patient-centered approach. Further research is imperative to clarify the optimal therapies for retinal migraine and the long-term outcomes.

## Author contributions

**Conceptualization:** Abdullah Alhewiti.

**Investigation:** Abdullah Alhewiti.

**Data curation:** Abdullah Alhewiti.

**Methodology:** Abdullah Alhewiti.

**Formal analysis:** Abdullah Alhewiti.

**Writing – original draft:** Abdullah Alhewiti.

**Writing – review & editing:** Abdullah Alhewiti.
